# Unified Electromagnetic-Electronic Design of Light Trapping Silicon Solar Cells

**DOI:** 10.1038/srep31013

**Published:** 2016-08-08

**Authors:** Javaneh Boroumand, Sonali Das, Abraham Vázquez-Guardado, Daniel Franklin, Debashis Chanda

**Affiliations:** 1 Department of Physics, University of Central Florida, 4111 Libra Drive, Physical Sciences Bldg. 430, Orlando, Florida 32816, USA; 2 NanoScience Technology Center, University of Central Florida, 12424 Research Parkway Suite 400, Orlando, Florida 32826, USA; 3 CREOL, The College of Optics and Photonics, University of Central Florida, 4304 Scorpius St., Orlando, Florida 32816, USA

## Abstract

A three-dimensional unified electromagnetic-electronic model is developed in conjunction with a light trapping scheme in order to predict and maximize combined electron-photon harvesting in ultrathin crystalline silicon solar cells. The comparison between a bare and light trapping cell shows significant enhancement in photon absorption and electron collection. The model further demonstrates that in order to achieve high energy conversion efficiency, charge separation must be optimized through control of the doping profile and surface passivation. Despite having a larger number of surface defect states caused by the surface patterning in light trapping cells, we show that the higher charge carrier generation and collection in this design compensates the absorption and recombination losses and ultimately results in an increase in energy conversion efficiency. The fundamental physics behind this specific design approach is validated through its application to a 3 μm thick functional light trapping solar cell which shows 192% efficiency enhancement with respect to the bare cell of same thickness. Such a unified design approach will pave the path towards achieving the well-known Shockley-Queisser (SQ) limit for c-Si in thin-film (<30 μm) geometries.

Low cost and high efficiency solar cells are an important alternative to fossil fuel based energy. Mono-crystalline silicon (c-Si) is undoubtedly the material of choice in photovoltaic applications due to its high natural abundance, low cost, reliability, excellent optical/electronic properties and chemical/radiation hardness^1^,^2^,^3^,^4^. However, there is continuous demand for thinner, lighter and more efficient solar cells. Present commercial screen-printed c-Si cells are typically 150–180 μm thick, enabling large absorption of the solar spectrum over the silicon bandwidth, and obtain 17–20% energy conversion efficiency in volume production^1^,^5^,^6^. While reducing the thickness of c-Si can decrease the absorption significantly for thickness below ~50 μm, it has been shown that it leads to a higher voltage^7^,^8^,^9^ with the added benefit of reduced material costs^10^,^11^,^12^. As the cell thickness decreases, the diode saturation current decreases, resulting in the increase in voltage. In order to compensate for the low absorption of ultra-thin (<30 μm) c-Si cells, special light management schemes were developed^13^,^14^,^15^,^16^,^17^. Various light trapping schemes were proposed and demonstrated in recent years to enhance absorption in thin-film geometry based on top and/or bottom diffractive/plasmonic light scattering patterns^18^,^19^,^20^,^21^,^22^,^23^,^24^,^25^,^26^. In our previous works we have shown light trapping in ultra-thin silicon solar cells and demonstrated significant enhancement in energy conversion efficiency in a functional cell for the first time^27^,^28^. However, light trapping solar cells must also ensure maximum charge carrier collection in order to fully benefit from the increase in photon absorption. Maximizing photon absorption without efficient electron collection is not adequate to obtain higher efficiency. An electronic device physics model which includes light trapping within functional cell geometries is missing from the present literature. Thus, there is a need for a unified photon-electron harvesting scheme that enables the design of high efficiency, functional, commercial grade solar cells. This will translate to enormous material cost savings (greater W/g silicon utilization) and provide other mechanical attributes such as light-weight and flexibility to the resultant solar modules. Here, we report a unified electromagnetic and electronic device physics design approach which maximizes the combined photon-electron harvesting. The spatial absorption profile of the light trapping solar cell predicted from finite difference time domain (FDTD) simulations define the charge carrier generation in the following electronic device simulation. The predicted short circuit current (J_sc_), open circuit voltage (V_oc_) and efficiency (η) from an optimized doping profile and carrier life time closely match experimental observations in bare as well as light trapping cell geometries.

## Results and Discussion

### Light trapping c-Si solar cell optical responses

**Figure 1:**
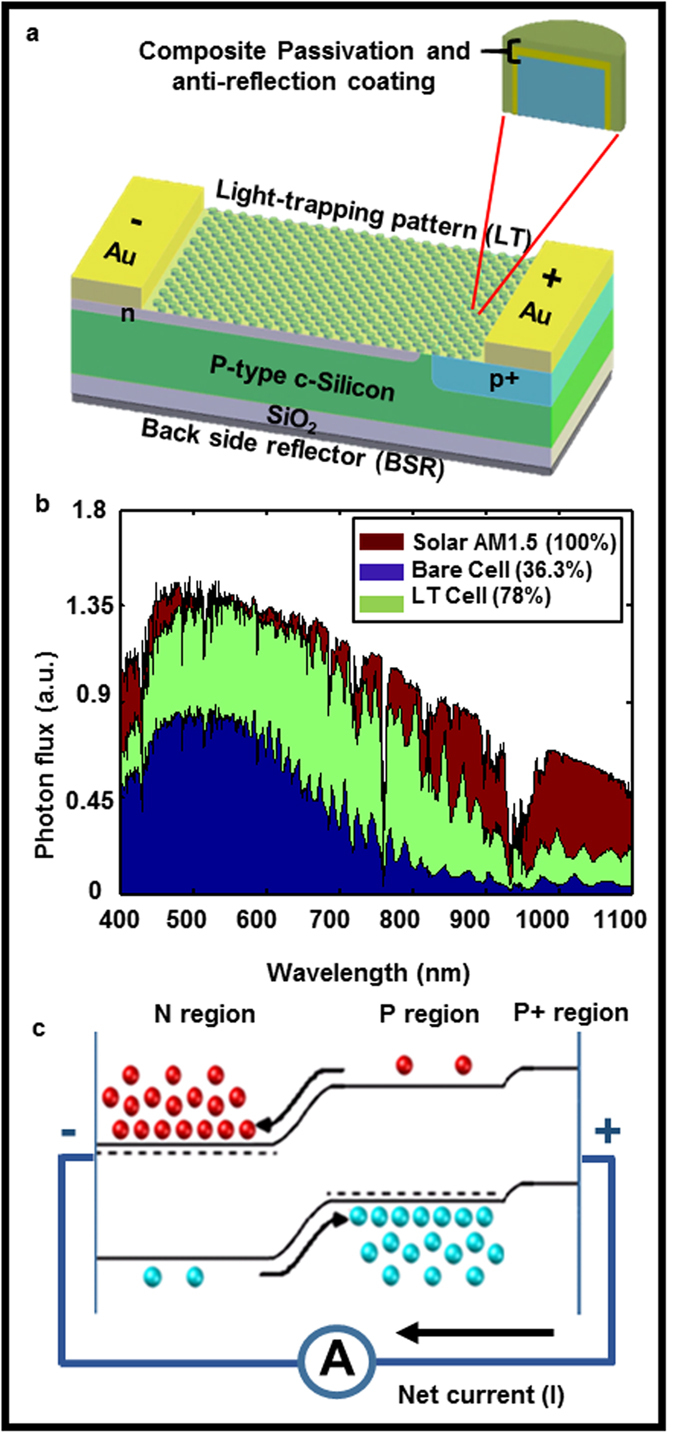
Device schematic, optical absorption, and band diagram. (**a**) Illustrates the c-Si solar cell architecture which combines the light trapping scheme with the functional cell geometry. (**b**) The absorbed photo flux as a function of wavelength with reference to AM1.5D solar spectrum for bare and light trapping cell inside a 3 μm thick wafer and (**c**) the corresponding band diagram under illumination.

Figure 1(a) shows the chosen light trapping cell architecture. The light trapping scheme is composed of top nanostructured diffractive optical element coated with anti-reflection coating coupled to an optical cavity which enhances absorption via a composite phenomenon: reduction in reflection, path length enhancement via forward diffraction and trapping light into the silicon waveguide and cavity modes. An integrated 78% absorption with respect to the AM1.5G spectrum is numerically predicted inside the 3 μm thick light trapping cell as shown in Fig. 1(b). This constitutes 117% absorption enhancement compared to a bare wafer of same thickness which absorbs only 36% of the integrated solar spectrum. For the top diffractive pattern a 2D hexagonal Bravais lattice was chosen based on the design reported in our earlier publications^27^,^29^. Figure 1(c) presents the band diagram of the corresponding n-p-p + junction under illumination when is connected to an external load. The lack of the acceptor impurity concentration at the base contact causes the minority carriers to recombine more easily, which is considered as one of the main sources of the voltage loss in a solar cell^19^. Hence, a heavily p-doped region at the base electrode improves the open circuit voltage. The band tilts downwards in the n region and upwards in the p region due to the positive and negative charge movement, respectively. Under illumination, the number of generated excess electrons is equivalent to the number of generated excess holes; hence the Fermi energy difference at the n and p sides, *ε*_*fn*_−*ε*_*fp*_ becomes closer to the band gap energy by increasing the numbers of excess minority carriers which leads to higher current density. This phenomenon is further described in the following section.

**Figure 2:**
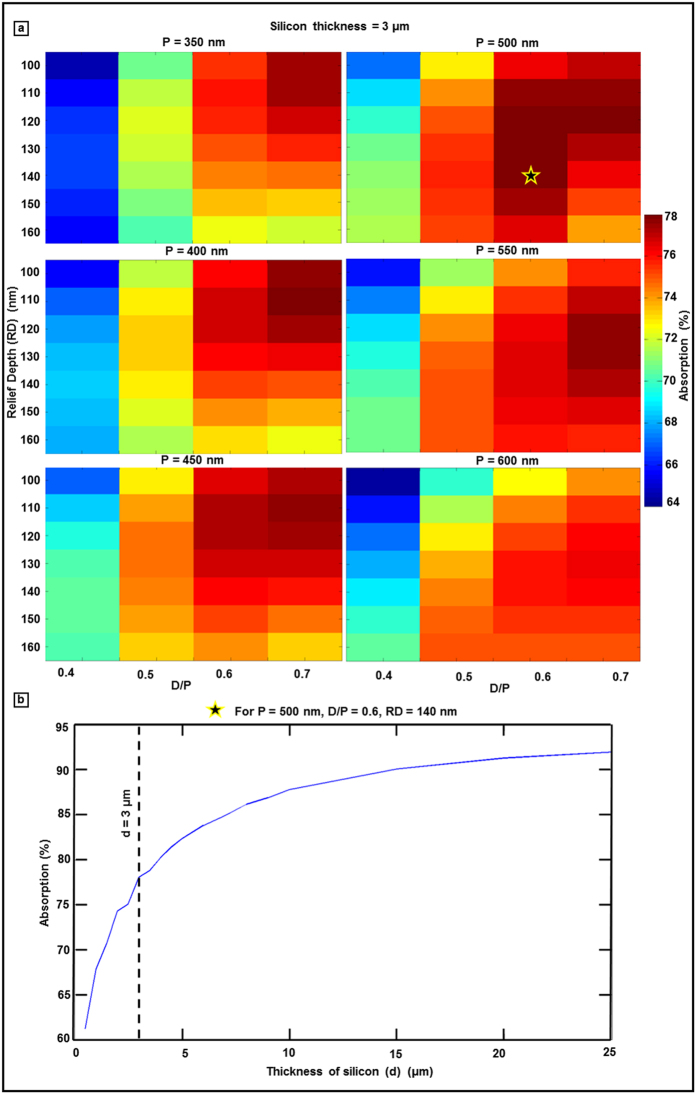
Light trapping pattern dimension and Si thickness optimization. (**a**) FDTD predicted light trapping pattern optimization for a constant silicon thickness (3 μm). The wavelength integrated absorption is maximized as a function of 2D hexagonal lattice period, D/P and relief depth. (**b**) Silicon absorption as a function of silicon thickness for the optimized light trapping pattern (P = 500 nm, D/P = 0.6, RD = 140 nm with ARC (SiO_2_/SiN = 50/35 nm)).

**Figure 3:**
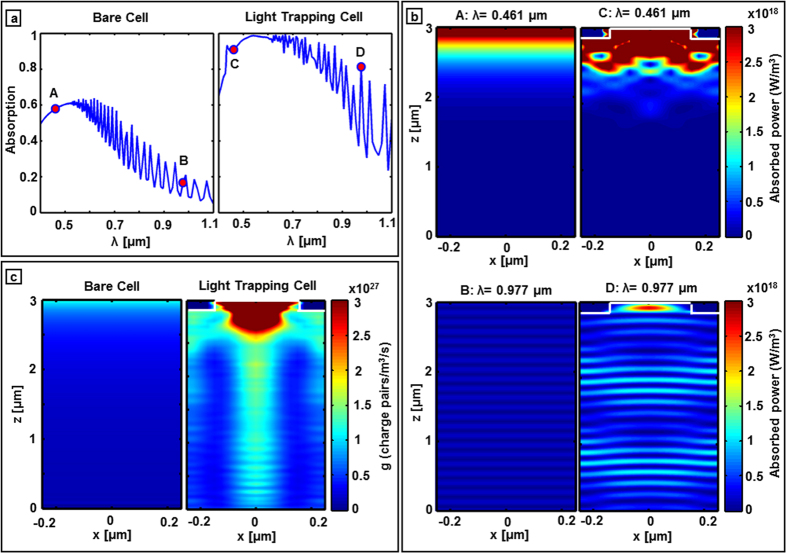
Optical characterization of the device. (**a**) Compares the FDTD predicted absolute absorption inside 3 μm thick bare and light trapping cells. (**b**) The power absorbed per unit volume in 3 μm thick bare and light trapping cell at strong (λ = 461 nm) and weak (λ = 977 nm) absorbing regimes. (**c**) Compares the wavelength integrated charge carrier generation rate (g) over a 2D plane across the center of the hexagonal unit cell.

Figure 2(a) shows the 3D FDTD predicted light trapping pattern optimization for a constant silicon thickness (3 μm). The wavelength integrated absorption is maximized as a function of 2D hexagonal lattice period (P), diameter to period ratio (D/P) and relief depth (RD) for a constant ARC layer (SiO_2_/SiN = 50/35 nm). According to Fig. 2(a), the optimized light trapping pattern which enables the maximum absorption of 78% integrated solar spectrum (400–1100 nm) (marked as star on Fig. 2(a)) defines the posts dimension of period 500 nm, diameter/period 0.6, and relief depth 140 nm. Further based on this optimized pattern, the silicon absorption as a function of silicon thickness is studied and shown in Fig. 2(b). It can be seen that for this light trapping design silicon thickness of >15 μm is needed in order to absorb >90% integrated AM1.5 solar spectrum. Figure 3(a) compares the FDTD predicted absolute absorption of a 3 μm thick bare and a light trapping cell as a function of wavelength. Significant absorption enhancement can be observed in the light trapping cell especially near the band edge where bare silicon absorption is weak. Furthermore, narrow Fabry-Perot cavity resonances are observed in the light trapping cell which correspond to the presence of the 0^th^ and higher order cavity modes of the thin cell. The FDTD predicted 2D absorption profiles (P_abs_) of bare and light trapping cells in a strong (λ = 461 nm) and weak (λ = 977 nm) absorption regimes are shown in Fig. 3(b). Absorbed power is obtained by applying equation (1), which is derived from the divergence of the Poynting vector formula as


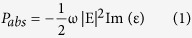


According to the equation (1), the energy absorption of a monochromatic light is directly proportional to the electric field intensity which mandates field enhancement for higher absorption. As predicted, the band edge photons (λ = 977 nm) are more strongly absorbed in the light trapping cell compared to the bare cell due to tight field confinement and path length enhancement via diffraction/scattering. There is significant enhancement in absorbed power between light trapping and bare cells which can be noticed by comparing Fig. 3(b), top and bottom. This fact is further supported by the wavelength integrated generation rate (g) in Fig. 3(c). The number of electron-hole pairs generated by absorbed photons as a function of position integrated over a specific wavelength range in the device is defined as the wavelength integrated charge carrier generation rate (g). From Fig. 3(c), significantly enhanced charge carrier generation can be observed for the light trapping cell due to the stronger photon absorption substantially deep inside the wafer. Subsequently, the FDTD predicted 3D generation rate data is imported into the numerical electronic modeling (Lumerical DEVICE, Lumerical Inc.) that makes the bridge between electromagnetic absorption and electronic device performance predictions. Since the actual cell is longer than one unit cell due to the lateral p-n junction (Fig. 1(a)), the FDTD predicted one unit cell generation rate, which is calculated with the periodic boundary condition along the x-y directions, is copied along the length of the device to cover the distance between two contacts.

### Electronic device modeling

**Figure 4:**
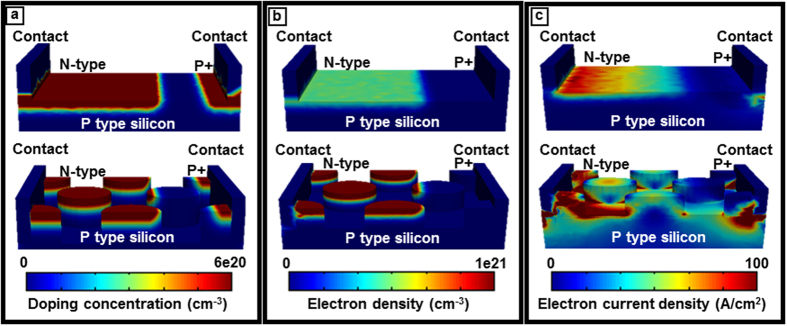
Doping and charge carrier generation profile. (**a**) The doping profile of the 3 μm thick silicon cell with and without light trapping. The p and n regions are defined which show the gradient of impurities in the device. (**b**) Compares the electron density of a 3 μm bare cell with light trapping cell. The electron density in n-type region of the light trapping cell is higher than that of the bare cell, and (**c**) shows the corresponding electron current density. The structure geometries in this figure are not to scale. The color bar upper and lower limits are chosen in order to enhance the contrast.

The quasi fermi energy levels, electron-hole densities and recombination losses of the cell were extracted from the electronic simulations in order to understand the fundamental gain and loss mechanisms. For a fair comparison, both bare and light trapping cells are assumed to be perfectly passivated with reasonably low surface recombination velocity (SRV) SRV = 10 m/cm. A low SRV isolates the effects of recombination and electron-hole generation processes. Figure 4(a) shows the chosen doping profile of the 3 μm silicon cell with and without light trapping. The p and n regions are shown as gradient of the doping concentration using two opposite color schemes. Figure 4(b) compares the electron density of a 3 μm bare and light trapping cells. The light trapping cell shows a higher absolute electron (hole) density due to the higher absolute quasi fermi energy levels which, in turn, originates from the higher absorption. The electron density in non-equilibrium conditions (i.e. under illumination as a function of the quasi fermi energy) is given by


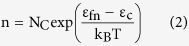


where 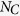 is the effective density of state in the conduction band, *ε*_*fn*_, and *ε*_*c*_ are electron quasi fermi energy and conduction band energy respectively. The carrier density is highly sensitive to any small variation in quasi fermi energy level. As can be seen in Fig. 4(b), the electron density within the n-type region of the light trapping cell is significantly higher than that of the bare cell. The n and p quasi fermi energy difference (*ε*_*fn*_ − *ε*_*fp*_) is a measure of the deviation from equilibrium. Under non-equilibrium conditions, i.e. under illumination, the current density can be written as


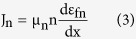


where *μ*_*n*_ is the electron mobility. The electron density in the non-equilibrium condition is n = n_0_ + Δn where n_0_ is the donor impurity concentration (at equilibrium) and Δn is the excess minority carrier concentration that is generated under illumination. The higher value of charge density, in conjunction with higher quasi fermi energy, leads to a greater electron current density. Figure 4(c) illustrates the electron current density enhancement in light trapping cell with respect to the bare cell. Current density that is proportional to the gradient of the quasi fermi level, shows the flow of photo generated carriers towards ohmic contacts and through the external circuit. The concentration of holes and corresponding hole current density can be explained in a similar fashion. For fair comparison, both electron density and current density (Fig. 4(b,c)) are integrated over the corresponding predicted I-V curve shown in the following.

**Figure 5:**
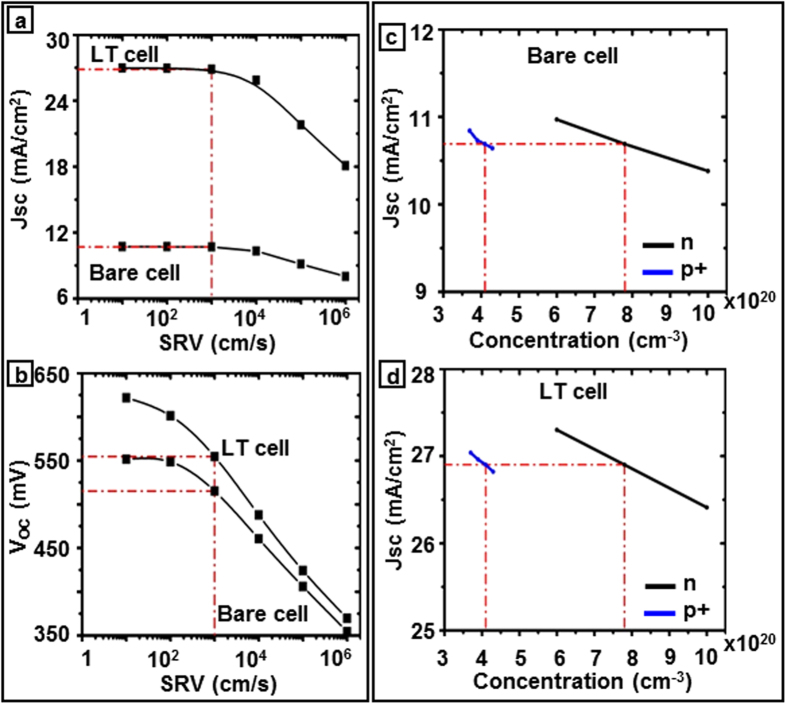
Effect of surface recombination and doping concentration. (**a,b**) The predicted short circuit current density (J_sc_) and open circuit voltage (V_oc_) as a function of surface recombination velocity in the top and bottom graphs respectively, and (**c,d**) show the predicted short circuit current density as a function of doping concentration for both bare and light trapping cells.

Apart from the light absorption enhancement in the light trapping cell, which leads to higher internal current density as demonstrated above, it is also important to investigate the mechanisms of loss in order to improve charge collection and overall cell efficiency. The surface recombination velocity (SRV), which is one of the main sources of voltage loss and low short circuit current, defines the carrier recombination rate at the silicon interfaces due to the silicon dangling bonds. Figure 5(a) shows the predicted variation in J_sc_ as a function of SRV for bare and light trapping cells. Although, a lower SRV (1–100 cm/s)^30^,^31^,^32^,^33^ produces higher J_sc_, achieving such low SRV on a patterned surfaces of a light trapping cell requires excellent surface passivation. The impact of bulk and surface recombination on the cell performance can be described by the effective lifetime, given by^30^,^34^,^35^


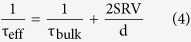


where d is the cell thickness. According to the equation (4), in lower cell thicknesses, SRV becomes more significant than the bulk lifetime. Thus a good multi-functional passivation layer on the patterned top surface which also functions as an anti-reflection coating makes a significant difference in device performance due to both photon absorption enhancement and carrier lifetime improvement. The SRV further influences the open circuit voltage which is defined in equation (5) for a solar cell made on a p-type wafer as^36^,^37^,^38^


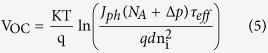


where J_ph_ is photocurrent density, N_A_ is the acceptor concentration, ∆n and ∆p are the electron and hole excess minority carrier concentration respectively which are equivalent under the illumination in solar cells, and n_i_ is the intrinsic carrier concentration. Since SRV is inversely related to the lifetime, τ_eff_ (Equation (4)), it reduces the open circuit voltage as can be observed in Fig. 5(b), and ultimately the energy conversion efficiency of the cell. Both the current density and the voltage (Fig. 5(a,b) of the light trapping cell decreases significantly compared to that of the bare cell as SRV increases. This sharp fall can be explained by higher surface recombination due to the surface texturing in light trapping cells. Figure 5(c,d) shows the short circuit current density vs doping concentrations for boron and phosphorus regions in bare and light trapping cells respectively for the surface recombination velocity of 1000 cm/s. A SRV of 1000 cm/s is chosen for the doping concentration studies since lower SRVs can only be obtained by thermal silicon oxide growth which is not applicable in this case due to polymeric substrates of fabricated microbar cells^29^,^39^. A significant enhancement in J_sc_ can be observed in the light trapping cell compared to the bare cell. The phosphorus and boron doping concentration studied regime is 6e + 20–1e + 21 cm^−3^ and 3.7e + 20–4.3e + 20 cm^3^, respectively. These variation ranges are based upon the pre-deposition furnace temperature of 900 °C–1050 °C in which the cells are exposed to the infinite constant sources^40^. Since our studied cell is ultrathin, 3 μm, compared to conventional silicon solar cell thickness (150–180 μm) the drive-in diffusion process that is occurred at higher temperature (T > 1000 °C) in the absence of the dopant source in order to increase the junction depth is not considered in this study. These results imply that lower doping concentration leads to higher current densities, at the studied doping concentration regime^41^ which also can be shown theoretically by:


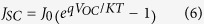


where J_0_ is the dark current which flows through the solar cell when a bias is applied in the dark and in low level injection is given by:


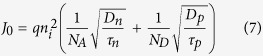


D_n_, D_p_ are the electron and hole diffusion coefficients, *τ*_*n*_, *τ*_*p*_ are the lifetime of electron and hole, respectively. Comparing Eq. (6) and Eq. (7) we can conclude that a lower doping concentration (N_A_, N_D_) leads to higher J_0_ and consequently higher J_sc_ as observed in the numerical simulation in Fig. 5(c,d).

**Figure 6:**
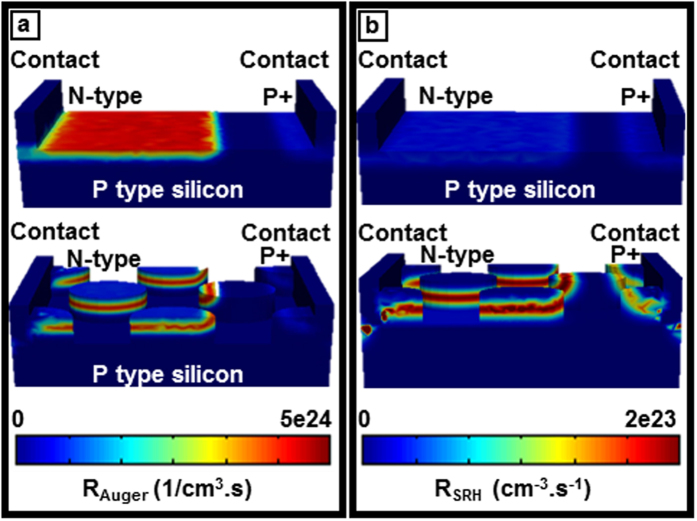
Recombination losses. (**a**,**b**) Compares the Auger and Shockley-Read-Hall recombination in bare and light trapping cells, respectively. Light trapping cell possesses higher recombination due to higher localized doping concentration and defect density. The cell geometry is not to scale.

**Figure 7:**
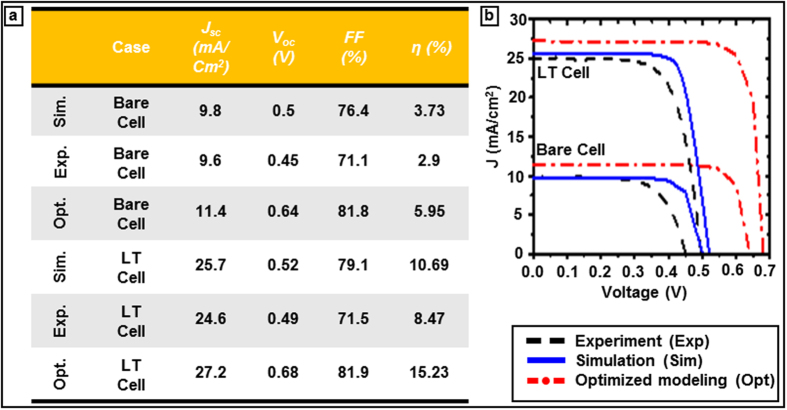
Comparison of the fabricated and predicted device performance. (**a**) Short circuit current (J_sc_), open circuit voltage (V_oc_), fill factor (FF), and efficiency (η) of fabricated, simulated and optimized 3 μm bare and light trapping solar cells and corresponding I-V curves are shown in (**b**).

Deep level or Shockley-Read-Hall (SRH) and Auger recombination are the other two carrier loss mechanisms which severely affect solar cell performance. The more heavily doped material leads to higher auger recombination and the material with more defect states has higher Shockley-Read-Hall recombination. These recombination mechanisms are defined as^42^:


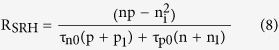


where 
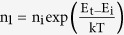
, 
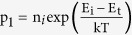
, τ_n0_, τ_p0_ are SRH electron and hole lifetime,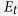 is trapping energy level, and:


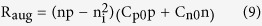


where Cp_0_, Cn_0_ are auger coefficients which have inverse quadratic dependence upon carrier concentration (unit cm^6^/s). From Fig. 6(a,b) it can be seen that the light trapping cell has a higher Auger and Shockley-Read-Hall recombination rate within the posts due to the generation of higher excessive minority carriers and higher defect density, respectively. Both recombination rates are integrated over the respective I-V curve (Fig. 7(b)) of bare and light trapping cells in order to demonstrate the overall response. The light trapping nano-patterning introduces more localized doping distribution and more defects to the silicon cell which leads to higher auger and SRH recombination. However, enhanced absorption and carrier generation in light trapping cells compensates these recombination losses and gives a higher overall cell efficiency. Further, this work shows that the device performance can be enhanced by minimizing recombination losses via optimum doping profile and surface passivation.

### Device optimization and simulation parameters

The device simulation parameters are presented in the Table (1) for two conditions: specific to the fabricated and the optimum cells. The values for the predicted cell were chosen to be either equivalent or close to the experimental conditions. There are some discrepancies between predicted and measured cell performances primarily due to the mismatch between estimated device parameters like carrier life time, SRV, sidewall ARC thickness on the patterned surface, doping profile and actual experimental cell. The detailed light trapping pattern design and fabrication is reported in our previous publications^29^. Soft nanoimprint lithography (NIL) and reactive ion etching defines hexagonal cylindrical silicon posts of period 500 nm, diameter 300 nm and relief depth 140 nm as light trapping pattern which are optimized with FDTD simulation as shown in Fig. 2(a). A layer of PECVD SiN_x_ (20–50 nm) and SiO_2_ (~50–80 nm) deposition the device serves as anti-reflection coating (ARC) and passivation layer. Different techniques of minimizing the reflection losses such as single dielectric layer, gradient refractive index layers, and nanostructured surfaces are commonly used^43^,^44^,^45^. A 200 nm thick gold layer serves as backside reflector (BSR). The top diffractive optics pattern functions as grating coupler to couple incoming solar radiation as diffracted/scattered modes thus enhancing effective path length. The functional cell fabrication is completed with the doping of boron and phosphorus through patterned hard masks to define the P-N junctions followed by metal contact formation as schematically shown in Fig. 1(a). These parameters are fixed for both bare and light trapping cells in order to compare and focus on the impact of light trapping on the device performance. The predicted results and experimentally measured data^28^ of a 3 μm thick microbar silicon solar cell are tabulated and plotted side by side for the comparison in Fig. 7(a,b), respectively. As shown in the table Fig. 7(a), the predicted J_sc_ and V_oc_ closely match experimental measurements for both bare and light trapping cells. The energy conversion efficiency enhancement prediction of 186% closely matches the experimentally measured enhancement of 192% of the light trapping cell with respect to the bare cell measurement. The main difference between the predicted and the fabricated cell is the V_oc_ that is most likely caused by non-uniform back surface field (BSF) of the fabricated cell. The optimized light trapping and bare cells show 105% and 80% percentages of improvements with respect to corresponding experimentally fabricated cells, respectively. The optimum cell design considered an ideal carrier life time and SRVs as tabulated in Table (1) in order to establish a maximum possible cell performance. For the optimized cell we used a SRV of 10 cm/s and bulk life time of 1 ms as tabulated in Table 1. Such range of low SRV and long carrier life time is indeed achievable via high quality surface passivation as demonstrated in refs 19,46,47. The 3D device simulation of charge carrier generation/recombination in bare and light trapping cells in thin-film geometry in this study distinctively showed the inter-relation between various processes. The study also defined parameter space (doping profile, junction depth, SRV etc.) for optimum performance of a 3 μm thick light trapping solar cell. The future work will focus on bridging the gap between the present and the optimal cell performances following this unified electromagnetic-electronic design approach presented above.

We theoretically demonstrated an optimized 3 μm thick light trapping cell in order to show what can be achieved in terms of device performance following this unified design approach. The light trapping scheme showed significant enhancement in band edge photon absorption resulting in higher charge carrier generation rate. Although surface nano-patterning causes more recombination, its impact on the overall cell efficiency enhancement is significant due to the stronger light absorption and higher generation rate which outweighs recombination losses. Such a unified electromagnetic-electronic design approach will help design better solar cell architecture with higher energy conversion efficiency.

## Additional Information

**How to cite this article**: Boroumand, J. *et al*. Unified Electromagnetic-Electronic Design of Light Trapping Silicon Solar Cells. *Sci. Rep*. **6**, 31013; doi: 10.1038/srep31013 (2016).

## Figures and Tables

**Table 1:** The electrical simulation parameters of the fabricated (Sim.) and optimized (Opt.) bare and light trapping cells.

	Wafer conc. (1/cm^3^)	p + surface conc. (1/cm^3^)	p + junc. width (nm)	n surface conc. (1/cm^3^)	n Junc. width (nm)	Electron Carrier lifetime (ms)	Hole Carrier lifetime (ms)	SRV (cm/s)	BSF conc. (1/cm^3^)	BSF junc. width (nm)
Opt.	10^15^	3.7E + 20	154	6E + 20	158	1	1	10	4.1E + 20	186
Sim.	10^15^	4.1E + 20	416	7.8E + 20	140	10^−3^	10^−4^	10^4^	4.1E + 20	186
